# Loss of ARPC1B impairs cytotoxic T lymphocyte maintenance and cytolytic activity

**DOI:** 10.1172/JCI129388

**Published:** 2019-11-11

**Authors:** Lyra O. Randzavola, Katharina Strege, Marie Juzans, Yukako Asano, Jane C. Stinchcombe, Christian M. Gawden-Bone, Matthew N.J. Seaman, Taco W. Kuijpers, Gillian M. Griffiths

**Affiliations:** 1 Cambridge Institute for Medical Research, University of Cambridge, Cambridge, United Kingdom.; 2 Department of Blood Cell Research, Sanquin Research and Landsteiner Laboratory, Academic Medical Center, University of Amsterdam, Amsterdam, Netherlands.; 3 Department of Pediatric Immunology, Rheumatology and Infectious Disease, Emma Children’s Hospital, Medical Center Amsterdam University, Amsterdam, Netherlands.

**Keywords:** Cell Biology, Immunology, Adaptive immunity, Cytoskeleton, T cells

## Abstract

CD8 cytotoxic T lymphocytes (CTLs) rely on rapid reorganization of the branched F-actin network to drive the polarized secretion of lytic granules, initiating target cell death during the adaptive immune response. Branched F-actin is generated by the nucleation factor actin-related protein 2/3 (Arp2/3) complex. Patients with mutations in the actin-related protein complex 1B (*ARPC1B*) subunit of Arp2/3 show combined immunodeficiency, with symptoms of immune dysregulation, including recurrent viral infections and reduced CD8^+^ T cell count. Here, we show that loss of ARPC1B led to loss of CTL cytotoxicity, with the defect arising at 2 different levels. First, ARPC1B is required for lamellipodia formation, cell migration, and actin reorganization across the immune synapse. Second, we found that ARPC1B is indispensable for the maintenance of TCR, CD8, and GLUT1 membrane proteins at the plasma membrane of CTLs, as recycling via the retromer and WASH complexes was impaired in the absence of ARPC1B. Loss of TCR, CD8, and GLUT1 gave rise to defects in T cell signaling and proliferation upon antigen stimulation of ARPC1B-deficient CTLs, leading to a progressive loss of CD8^+^ T cells. This triggered an activation-induced immunodeficiency of CTL activity in ARPC1B-deficient patients, which could explain the susceptibility to severe and prolonged viral infections.

## Introduction

The actin cytoskeleton coordinates a wide variety of cell functions ranging from cell division and differentiation to migration. The ability to reorganize the actin network in response to both extracellular and intracellular cues is pivotal for many cell types to perform their physiological functions (1). This is particularly relevant for cells of the immune system, including cytotoxic T lymphocytes (CTLs), which patrol the body in order to seek and destroy virally infected and transformed cells.

Cytolytic activity occurs upon CTL recognition of an antigen-presenting target cell via their TCR, which initiates the formation of a tight contact between the CTLs and target known as the immunological synapse (synapse). The initiation, formation, and maintenance of the synapse rely upon the polymerization and dynamic rearrangement of the cortical actin cytoskeleton (2, 3). Polarized secretion of granules by CTLs is intricately coordinated by filamentous actin depletion and recovery at the synapse, supporting a central role of actin in CTL effector responses (4–6).

Cortical actin comprises a network of branched-actin filaments largely nucleated by the actin-related protein 2/3 (Arp2/3) complex. This complex contains 7 subunits named Arp2, Arp3, and actin-related protein complex 1 (ARPC1), ARPC2, ARPC3, ARPC4, and ARPC5. Arp2 and Arp3 act as monomers from which new actin filaments elongate in a Y-branch orientation and recruit and hydrolyze ATP (7, 8). The other subunits (ARPC1–5) organize to form the structural core of the complex, providing an interface for binding to preexisting actin filaments and regulatory cofactors (9, 10). Optimal activity of Arp2/3 requires both the presence of ATP and engagement by nucleation promoting factor (NPF) proteins, including Wiskott-Aldrich syndrome (WAS) protein (WASp) and WASp-family verprolin-homologous protein (WAVE) (11). WASp and WAVE are engaged in distinct signaling pathways downstream of TCR activation, leading to Arp2/3-mediated F-actin initiation and maintenance, respectively (12). Another member of the WASp NPF family is WASp and SCAR homologue (WASH), which cooperates with Arp2/3 to regulate endosomal protein sorting, morphology, and receptor trafficking (13–15).

The localization of NPF proteins can focus Arp2/3 activity to distinct compartments within the cell, e.g., plasma membrane, cytoplasm, endocytic vesicles, or nucleus. This can give rise to specialized subcellular functions for Arp2/3 within the cell (16). In human cells, there are 2 isoforms of the ARPC1 and ARPC5 subunits, named ARPC1A, ARPC1B, ARPC5, and ARPC5L, respectively, that can be incorporated into the Arp2/3 complex, resulting in 4 different subfamilies. Functionally, Arp2/3 complexes containing the ARPC1B/ARPC5L combination have higher actin nucleating activity and produce filaments that are much more stable than the ARPC1A/ARPC5 combination. This has led to the notion that Arp2/3 complex composition dictates actin filament dynamics and ultimately Arp2/3 intracellular functions (17). Despite increasing evidence for the crucial role played by the Arp2/3 complex in many actin-dependent processes, the extent of its contribution to CTL functions has yet to be defined. How Arp2/3 activity regulates actin reorganization and directs granule secretion during CTL killing is not currently known. The recent identification of immunodeficient patients lacking ARPC1B (18–22) provides an opportunity to examine the role of Arp2/3 in CTLs, the contribution of CTLs to the immunodeficiency, and the molecular basis of the disease. Although alterations in actin polymerization explain a number of the defects in cell motility and platelet secretion, many aspects of the immunodeficiency reported in patients with mutations in ARPC1B are poorly understood, including the low CD8^+^ T cell counts and recurrent viral infections suggestive of loss of cytolytic activity.

Here, we report that Arp2/3 activity is pivotal for the cytolytic function of CTLs. TCR activation, degranulation, and target cell killing are all reduced in ARPC1B-deficient CTLs. Furthermore, we find that ARPC1B is required for selective receptor recycling to the plasma membrane via the retromer and WASH complexes. These include CD8 and TCR, 2 receptors critical for T cell signaling, as well as GLUT1, the major glucose transporter in T cells, which is essential for T cell proliferation. These results provide a molecular explanation for the decreased CD8 count and loss of CTL-mediated killing giving rise to increased susceptibility to acute as well as the more chronic viral infections, as observed in ARPC1B-deficient patients.

## Results

### The Arp2/3 complex colocalizes with actin at the immune synapse in CTLs.

**Figure 1:**
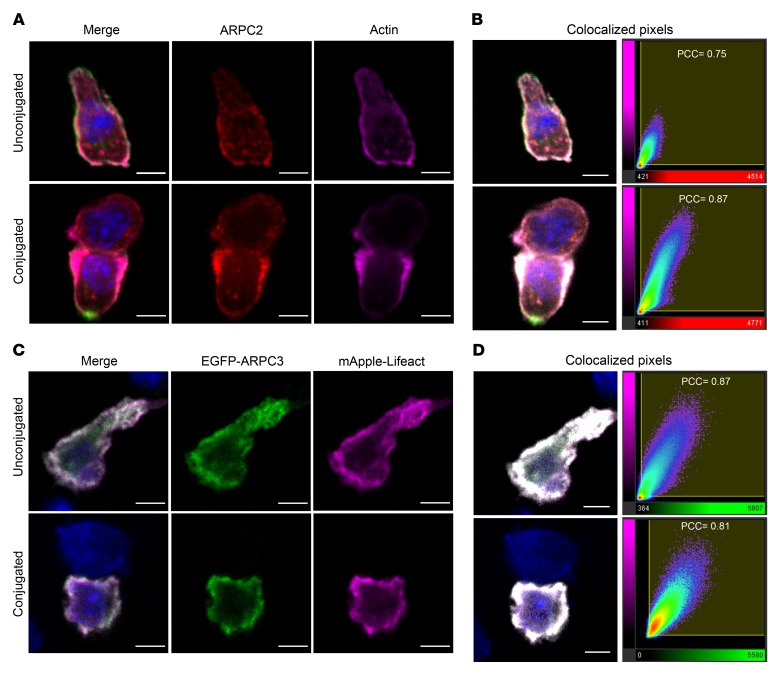
Arp2/3 colocalizes with actin in migrating and synapse-forming CTLs. (**A**) Single confocal slices of untransfected OT-I CTLs stained with antibodies against CD8 (green), ARPC2 (red), and actin (magenta). (**B**) Left panels: merged images from **A** illustrating the colocalized regions in white saturation after analysis. Right panels: colocalization graphs of ARPC2 voxels (red *x* axes) plotted against actin voxels (magenta *y* axes). (**C**) Single confocal slice of OT-I CTLs transfected with EGFP-ARPC3 (green) and mApple-Lifeact (magenta) constructs. (**D**) Left panels: merged images from **C** showing the colocalized regions in white saturation after analysis. Right panels: colocalization graphs of EGFP-ARPC3 voxels (green *x* axes) plotted against mApple-Lifeact voxels (magenta *y* axes). Conjugated cells were fixed 25 minutes after mixing with OVA-loaded EL4 target cells. Numbers on the graphs indicate the degree of colocalization expressed as a PCC. Nuclei stained with Hoechst (blue). Scale bars: 4 μm. Data representative of 2 independent experiments. (**A**) CTLs = 75; conjugates = 48. (**C**) CTLs = 66; conjugates = 44).

To address the role of the ARP2/3 complex in CD8 CTLs, we made use of the well-characterized OT-I transgenic mouse model in which all T cells express the same TCR that is specific for the ovalbumin peptide SIINFEKL (OVA_257–264_) (23). To localize the Arp2/3 complex, we labeled OT-I CTLs before and after synapse formation using an antibody against the ARPC2 subunit of the Arp2/3 complex (Figure 1, A and B). ARPC2 was evenly associated with the plasma membrane in OT-I CTLs prior to conjugate formation, colocalizing with F-actin with a few small puncta within the cytoplasm. Both ARPC2 and F-actin reorganized upon synapse formation with target cells, accumulating at the edges of the synapse and depleting from the center (Figure 1A). Image analysis revealed a high degree of colocalization between ARPC2 and actin, with a Pearson’s correlation coefficient (PCC) of 0.75 and 0.87 in unconjugated and conjugated OT-I CTLs, respectively (Figure 1B). A similar analysis of Arp2/3 localization in OT-I CTLs transiently expressing EGFP-tagged ARPC3 and mApple-Lifeact also showed ARPC3 colocalized with F-actin both before and after synapse formation (Figure 1, C and D; PCC = 0.87 and 0.81, respectively). These results demonstrate the recruitment of Arp2/3 at the synapse, suggesting that the Arp2/3 complex may play a role in regulating actin dynamics during synapse formation by CTLs.

### Optimal CTL effector function relies on active Arp2/3 complex.

**Figure 2:**
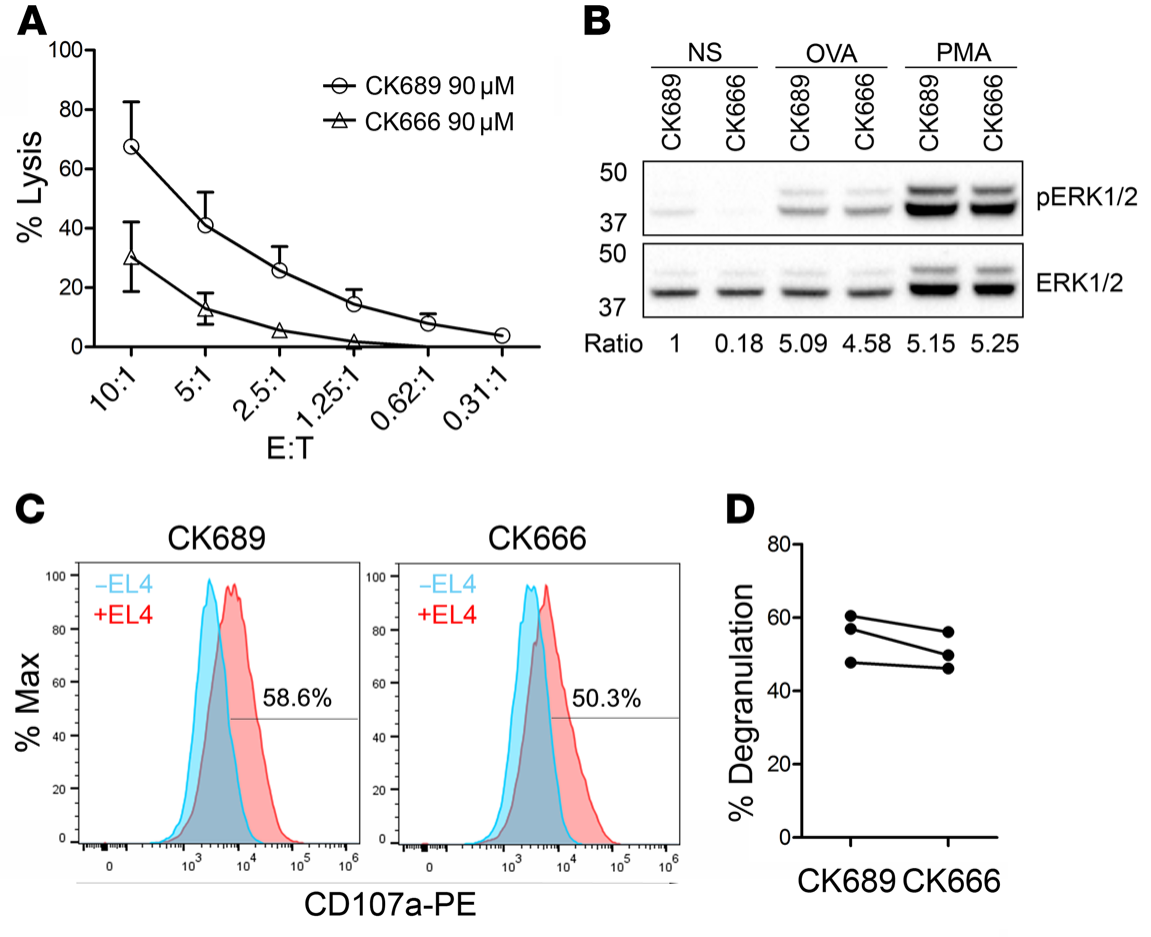
Arp2/3 inhibition affects CTL killing. (**A**) Killing capacity of OT-I CTLs treated with the inactive control compound CK689 or the Arp2/3 inhibitor CK666, expressed as a percentage of target cell lysis at the effector-to-target (E:T) ratios indicated (mean of 3 independent experiments; error bars indicate SEM). (**B**) Western blot of p-ERK1/2 and total ERK1/2 in nonstimulated (NS) cells or following stimulation with 1 μM OVA peptide or 50 nM PMA (for 15 minutes) in control versus treated cells (representative of 3 independent experiments). Numbers indicate the fold change (ratio) of p-ERK1 expression following stimulation and after normalization to total ERK1 expression. (**C**) Representative flow cytometry plot and quantitation (**D**) of LAMP1-PE (CD107a) uptake in OT-I CTLs in the absence (blue) or presence (red) of OVA-loaded EL4 (gated on CD8^+^ cells) after 3 hours following treatment with CK689 or CK666 (*n* = 3 independent experiments in duplicate).

The development of small compounds to inhibit Arp2/3 activity has provided a versatile tool to study Arp2/3-related functions in many cell types (24). CK666 is a reversible molecule that works by maintaining the complex in an inactive state, thereby preventing the nucleation of new actin filaments (25). To gain insights into the contribution of Arp2/3 in CTL effector functions, we assessed OT-I CTL–mediated killing in the presence of either the inactive compound CK689 or the inhibitor CK666. Treatment with CK666 led to a greater than 50% reduction in target cell lysis compared with treatment with the control compound CK689 (Figure 2A). We noted that CK666 treatment reduced the basal level of p-ERK in CTLs (Figure 2B), but made no difference to ERK phosphorylation triggered by TCR activation via high-dose antigen (OVA) or phorbol 12–myristate 13-acetate (PMA). We also found that, although target cell lysis was decreased upon inhibition of Arp2/3, we observed only a modest reduction in degranulation in response to OVA-loaded target cells (Figure 2, C and D). These results suggest a role for Arp2/3 in CTL-mediated killing that is independent of granule release.

### Arp2/3 activity controls actin remodeling at the synapse.

**Figure 3:**
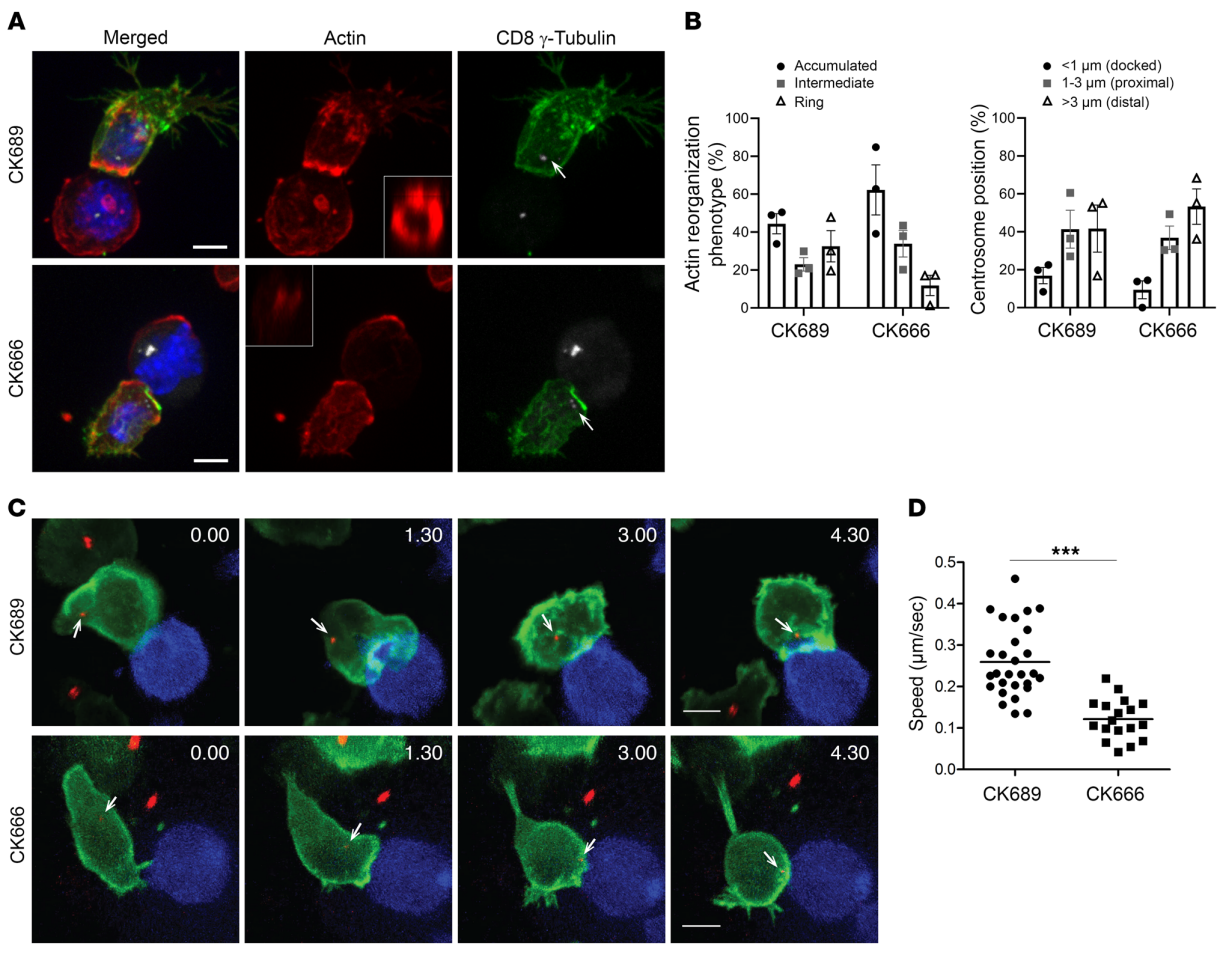
Arp2/3 inhibition impairs actin dynamics in CTLs. (**A**) Confocal projections of OT-I CTLs treated with 90 μM of CK689 or CK666 mixed with OVA-loaded EL4 for 25 minutes; cells were fixed and labeled with antibodies against CD8 (green), actin (red), and γ-tubulin (white) to mark the centrosome (white arrows). A representative 3D reconstruction of en face interaction (white box) of the actin phenotype at the interface between the OT-I CTLs and its target. Scale bars: 5 μm. (**B**) Quantitation derived from images as exemplified in **A** and showing the percentages of conjugates displaying the different actin reorganization phenotypes at the synapse (left panel, see Supplemental Figure 1) or the centrosome distance relative to the synapse (right panel: CK689 CTLs = 190, conjugates = 91, CK666 CTLs = 179, conjugates = 80, mean of 3 independent experiments, error bars indicate SEM). (**C**) Actin dynamics and centrosome position (white arrows) in OT-I CTLs expressing EGFP-Lifeact (green) and PACT-mRFP (red) during interaction with EL4 blue at various time point (min:s) from the first contact. Images are confocal projections from Supplemental Videos 1 and 2. Scale bars: 5 μm. (**D**) OT-I CTL motility while migrating on ICAM-1 following treatment with 90 μM CK666 or CK689. The speeds of cell migration were analyzed with time-lapse images (see Methods). The graph includes plots of 28 cells for CK689 and 18 cells for CK666 from 3 videos each. ***P < 0.0001, unpaired *t* test.

Target cell killing involves secretion of lytic granules requiring both actin depletion and centrosome docking at the synapse (5, 26). We asked whether actin depletion and centrosome polarization were disrupted when Arp2/3 was inhibited. Using quantitative microscopy, we evaluated the distribution of actin at the interface between mouse OT-I CTLs and EL4 target cells and measured the position of the centrosome relative to the synapse (Figure 3, A and B). OT-I CTL target conjugates were labeled using antibodies against F-actin, γ-tubulin, and CD8 (which is expressed by CTLs, but not by target cells) (Figure 3A). 3D reconstructions of each conjugate were used to examine actin across the synapse, and the actin localization was quantitated as described in Methods and Supplemental Figure 1 (supplemental material available online with this article; https://doi.org/10.1172/JCI129388DS1). In CK689-treated (control) OT-I CTLs, 50% of the conjugates showed actin accumulated across the synapse; 30% showed depletion of actin across the center of the synapse, with accumulation at the periphery resulting in a typical ring shape when visualized en face (Figure 3A and Supplemental Figure 1), and 20% of conjugates showed an intermediate phenotype with some depletion from the center of the synapse (Figure 3B and Supplemental Figure 1). Actin depletion across the center of the synapse was reduced upon CK666 treatment, with only 10% of conjugates showing actin depletion (Figure 3B and Supplemental Figure 1). The polarization of the centrosome toward the synapse was reduced upon CK666 inhibitor treatment, with 53% of conjugates showing the centrosome distal (>3 μm), compared with 40% after CK689 treatment (Figure 3B). We also examined actin dynamics at the synapse using live cell imaging, with OT-I CTLs expressing EGFP-Lifeact and the centrosome marker PACT-mRFP in the presence of CK689 or CK666 (Figure 3, C and D, Supplemental Video 1 and 2). Still images from the videos show a striking difference in the actin reorganization from first contact (0:00) in the presence of CK666, with reduced actin accumulation and depletion (Figure 3C) compared with CK689. OT-I CTL migration was also affected upon Arp2/3 inhibition (Figure 3D, Supplemental Videos 3 and 4). Control OT-I CTLs exhibited typical lamellipodia that ruffled extensively in migrating cells (Supplemental Video 3). In contrast, OT-I CTLs treated with CK666 were devoid of lamellipodia, but rather displayed small membrane blebs (Supplemental Video 4) and reduced migratory capacity, which was reflected in a significantly lower speed of motility compared with that of control OT-I CTLs (Figure 3D). These results suggest important roles for Arp2/3 during several stages leading to CTL killing, including cell motility, actin depletion across the synapse, and centrosome polarization to the synapse.

### Characterization of human CTLs lacking the Arp2/3 complex subunit ARPC1B.

**Figure 4:**
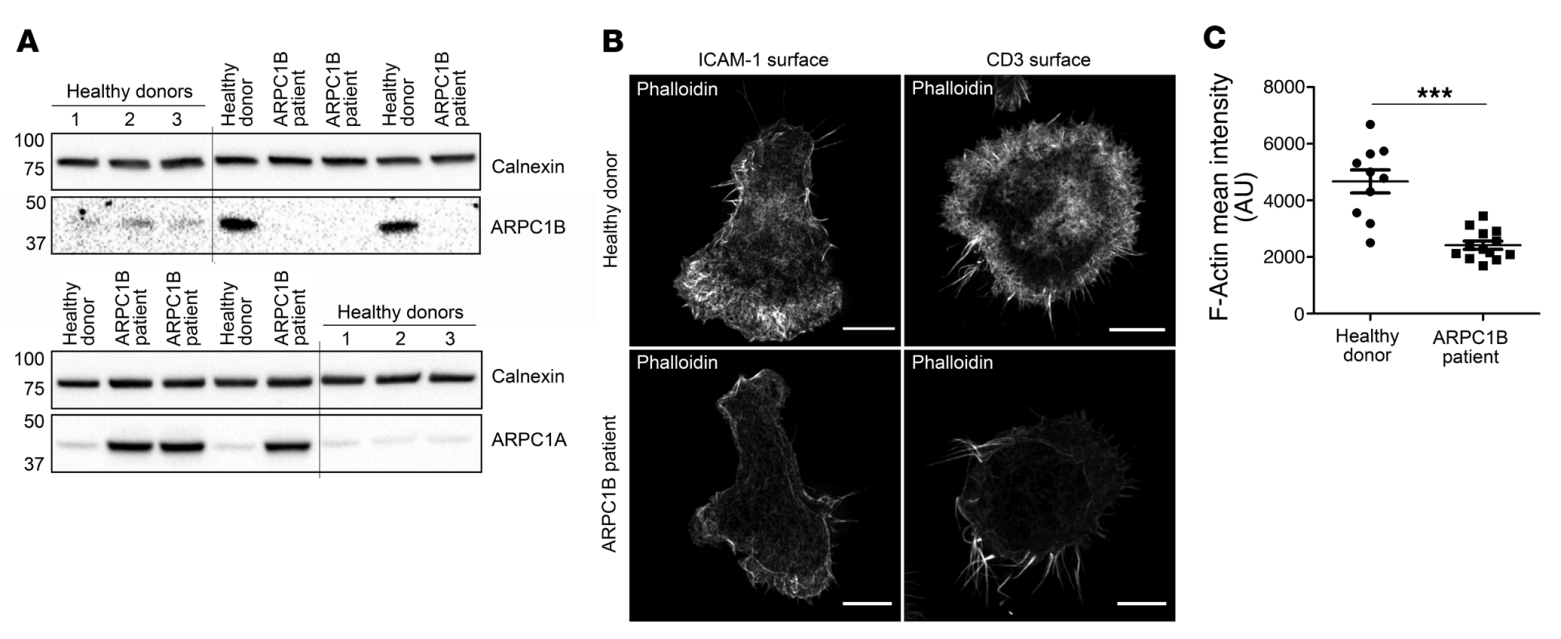
Differential actin structure in hCTLs from ARPC1B-deficient patient. (**A**) Immunoblot analysis of calnexin, ARPC1B, and ARPC1A in hCTL lysates from 4 different HD (lanes 1 to 4) and ARPC1B patient samples from day 20 (lanes 4 to 6) and day 12 (lanes 7 and 8) after expansion. HD 4 (lanes 4 and 7) was used for the rest of the study. MW markers in kD. Lanes were run on the same gel, but were noncontiguous. (**B**) Single plane of a *Z*-stack across hCTLs from HD or ARPC1B patient plated on ICAM-1– (0.5 μg/mL) or anti-CD3–coated (10 μg/mL; right panel) slide, fixed, stained with phalloidin and CD8 (not shown), and imaged using Structural Illumination Microscopy. Scale bars: 3 μm. (**C**) Quantitation of the mean intensity of phalloidin staining (F-actin) in HD or ARPC1B-deficient patient hCTLs generated from images as exemplified in **B** (right panel) (see Methods for description). HD *n* = 10 cells ARPC1B-patient *n* = 13 cells. ***P = 0.0053, unpaired *t* test. Data representative of 3 independent experiments.

We next asked whether these findings might also be apparent in the first ARPC1B-deficient patient that had been identified (18). Therefore, we differentiated human CTL (hCTL) expanded from a peripheral blood sample from this patient (see Methods). Western blot analysis of hCTL lysates from this patient showed a complete loss of ARPC1B together with an upregulation of the ARPC1A isoform when compared with lysates from healthy donor (HD) hCTLs (Figure 4A), in agreement with findings in activated T cells, EBV-transformed B cells, and neutrophils (22). Superresolution microscopy of phalloidin-labeled HD hCTLs (to image F-actin) showed thick lamellipodia at the leading edge in cells migrating on ICAM-1–coated surfaces, which appeared as a peripheral ring of dense, branched actin in hCTLs spread on anti-CD3–coated surfaces (Figure 4B). In contrast, ARPC1B-deficient hCTLs were devoid of lamellipodia, with an increased number of long filopodia-like projections emanating from a sparse actin network that failed to reorganize upon TCR activation (Figure 4B). These prominent filopodia were previously observed in ARPC1B-deficient neutrophils and platelets (18, 19) and were shown to be nucleated by formin activity (21). Such a shift in favor of formin-mediated nucleation is often noticed upon deletion of Arp2/3 components owing to the competition between Arp2/3 and formin for the pool of actin monomers (27–29). Furthermore, the overall intensity of the phalloidin staining in hCTLs from the ARPC1B-deficient patient was decreased relative to HD hCTLs, indicating a defect in actin polymerization following stimulation (Figure 4C). These results show that, despite the upregulation of ARPC1A, the loss of ARPC1B results in a disruption of the actin cytoskeleton in hCTLs, suggesting a critical role of ARPC1B in the activity of the Arp2/3 complex in hCTLs.

### ARPC1B-deficient hCTLs have modified and unstable immune synapses.

**Figure 5:**
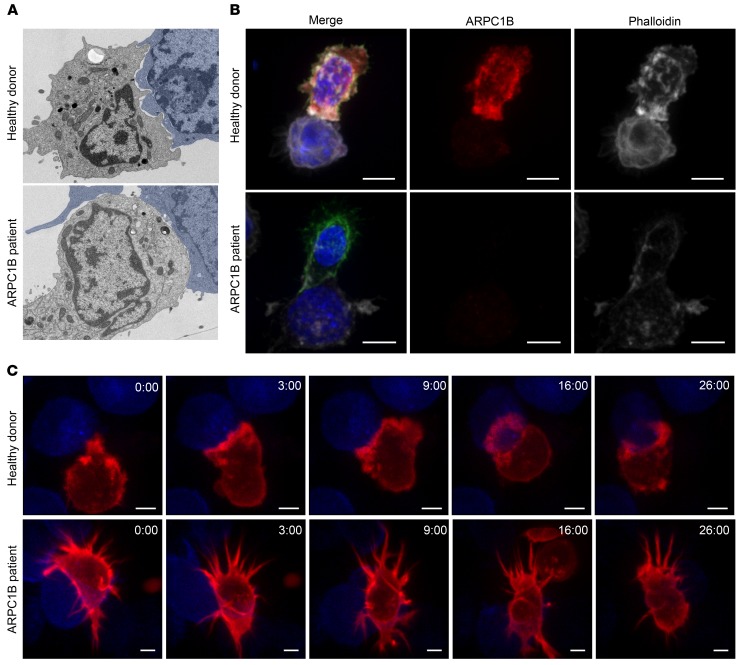
Electron and light microscopy reveal atypical synapse formation by ARPC1B-deficient hCTLs. (**A**) Electron micrograph of HD and ARPC1B patient hCTLs conjugated to P815 target cells (false colored in blue) for 25 minutes. (**B**) hCTLs from HD and ARPC1B-deficient patient were conjugated to blue P815 target cells, fixed, and labeled with anti-CD8 (green) and anti-ARPC1B (red) antibodies and phalloidin (white). 3D reconstructions of *Z*-stack are shown. Scale bars: 3 μm. Data are representative of 3 independent experiments (**A** and **B**). (**C**) Actin dynamics in HD and ARPC1B-deficient patient hCTLs expressing mApple-Lifeact (red) during interaction with blue P815 target cells. Images are single confocal slice from Video 3 at the indicated time points (min:s) from the first contact. Data are representative of 5 independent experiments. Scale bars: 5 μm.

The immunological synapse formed between CTLs and target cells involves extensive remodeling of the cortical actin cytoskeleton (30, 31). Given the defects in actin reorganization we had observed, we examined synapses formed by ARPC1B-deficient hCTLs with target cells by electron microscopy (Figure 5A). Many interdigitations were evident during early contact between HD hCTLs and target cells. By contrast, ARPC1B-deficient hCTL synapses were devoid of interdigitations, with membranes appearing more flattened. These differences were also apparent from immunofluorescence imaging of hCTLs labeled with antibodies against ARPC1B and phalloidin (which labels F-actin). ARPC1B-deficient hCTLs showed loss of ARPC1B and a reduced signal for actin with no actin accumulation at the synapse, while HD hCTLs showed strong signals for both actin and ARPC1B with an accumulation of actin at the synapse (Figure 5B). Live cell imaging of HD and ARPC1B-deficient hCTLs transduced with lentivirus expressing the F-actin probe mApple-Lifeact further illustrated the striking differences between the HD and the patient cells (Figure 5C, Supplemental Videos 5 and 6). HD hCTLs showed extensive actin-rich ruffles upon contact with target cells, resulting in actin accumulation 3:00 minutes after initial contact (Figure 5C), followed by actin depletion at the synapse center 16:00 minutes after initial contact (Figure 5C). ARPC1B-deficient hCTLs showed prominent filopodia, but neither actin accumulation nor depletion could be seen across the synapse (Figure 5C and Supplemental Video 6). Furthermore, HD hCTLs remained firmly attached to the target for a considerable period of time (26 minutes) (Supplemental Video 5), whereas ARPC1B-deficient hCTLs failed to do so within the same period of time. Taken together, these observations suggest that ARPC1B expression is required for Arp2/3-mediated actin remodeling at the synapse in hCTLs.

### Pleiotropic defects in ARPC1B-deficient hCTLs.

**Figure 6:**
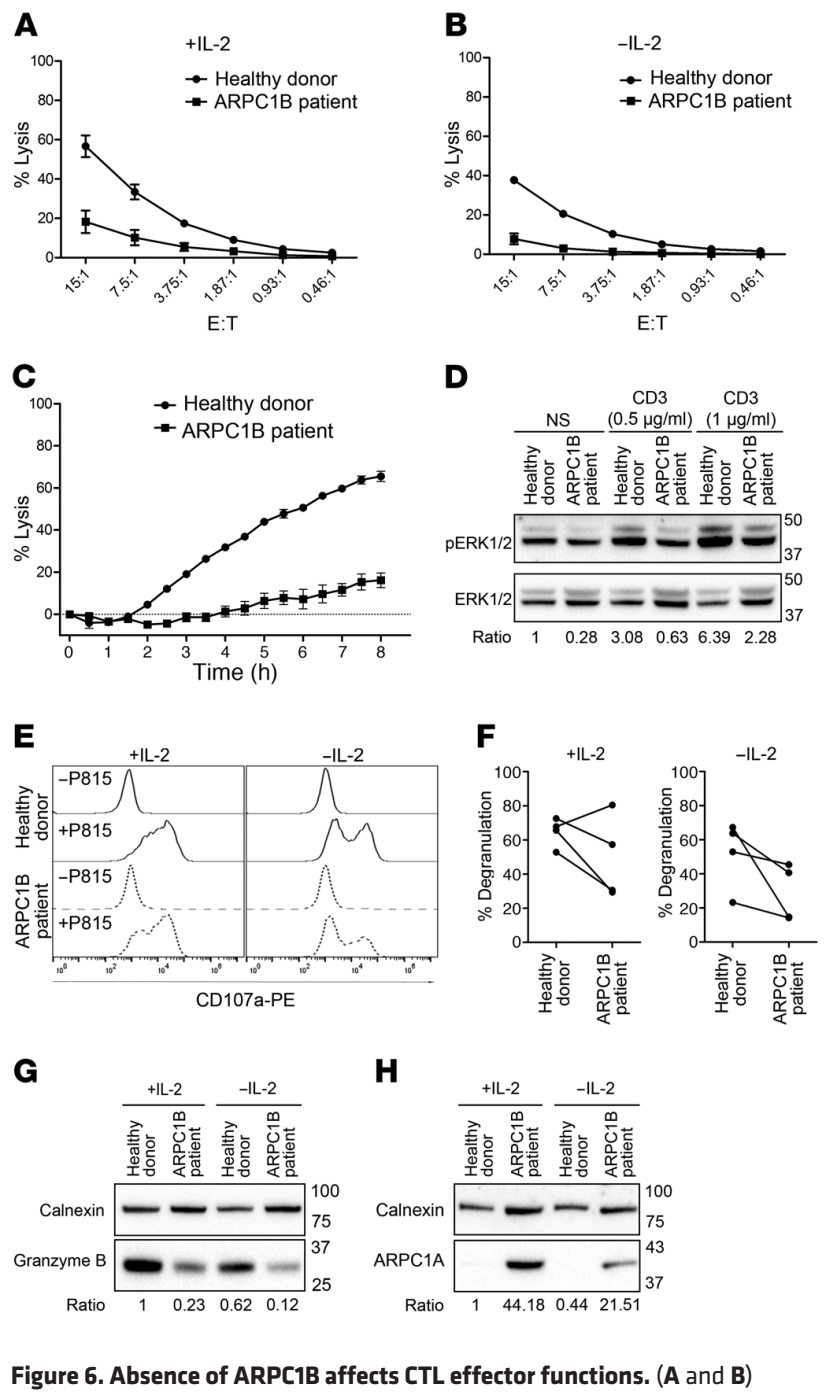
Absence of ARPC1B affects CTL effector functions. (**A** and **B**) Percentage lysis of P815 (after 2 hours) by HD or ARPC1B-deficient patient hCTLs at the indicated effector-to-target ratios in the presence (**A**) or absence (**B**) of IL-2. (**C**) Percentage lysis of P815-NucLight Red targets over time measured by IncuCyte killing assay using HD or ARPC1B-deficient patient hCTLs that have been deprived of IL-2 for 16 hours. (**D**) Western blot analysis of p-ERK1/2 and ERK1/2 expression in HD and ARPC1B-deficient patient hCTLs stimulated for 15 minutes with plate-bound anti-CD3 antibody at the indicated concentrations. Numbers indicate the fold change (ratio) of p-ERK2 expression following stimulation in ARPC1B-deficient patient and HD after normalization to total ERK2 expression. (**E**) Representative flow cytometry plot and quantitation (**F**) of LAMP1-PE (CD107a) uptake in HD and ARPC1B-deficient patient hCTLs (gated on CD8^+^ cells) after 2 hours incubation under the indicated conditions. (**G** and **H**) Immunoblot of granzyme B (**G**) and ARPC1A (**H**) in HD and ARPC1B-deficient patient in the presence or absence of IL-2. MW markers in kD. (**A**–**C**) Data are shown as mean of 3 independent experiments; error bars represent SEM. (**D**, **G**, **H**) Data are representative of 4 independent experiments.

Our results using the mouse OT-I system suggest that optimal killing of target cells requires Arp2/3 activity (Figure 2). Hence, we sought to examine the impact of ARPC1B deficiency on the effector functions of hCTLs. Previous work has shown that the presence of IL-2 during in vitro culturing of hCTLs or NK cells can restore cytotoxicity in hCTLs from familial hemophagocytic lymphohistiocytis 5 (FHL5, due to STXBP2 defect) or WAS patients (32–34). We therefore tested cytotoxicity and degranulation using hCTLs either cultured in IL-2 continuously or deprived of IL-2 for 16 hours prior to the lysis assay. Measuring LDH release during target cell lysis showed reduced cytotoxicity from ARPC1B-deficient hCTLs (20%) compared with HD hCTLs (58%, Figure 6A). Removal of IL-2 reduced cytotoxicity further for both HD and ARPC1B-deficient hCTLs (Figure 6B). Using the IncuCyte system to monitor loss of target cells over a longer time scale after IL-2 deprivation, it was clear that ARPC1B-deficient hCTLs failed to kill target cells, while HD hCTLs killed increasing numbers of targets over time (Figure 6C).

Western blot analysis of ERK phosphorylation in hCTLs showed reduced p-ERK in ARPC1B-deficient compared with HD hCTLs both under basal conditions and following TCR activation with 2 different concentrations of anti-CD3 (Figure 6D). Consistent with the reduced cytotoxicity and TCR signaling, upon target cell challenge, ARPC1B-deficient hCTLs showed a reduced mean percentage degranulation (56%) compared with HD hCTLs (71%, Figure 6, E and F). Upon IL-2 deprivation for 16 hours, degranulation was diminished in both HD (63%) and ARPC1B-deficient (40%) hCTLs in line with the reduced target cell lysis seen without IL-2 (Figure 6, E and F). Furthermore, granzyme B protein expression, which is known to be IL-2 dependent (35, 36), was also reduced in the absence of IL-2 with ARPC1B-deficient hCTLs compared with HD hCTLs (Figure 6G). Of note, the higher expression of ARPC1A isoform observed in ARPC1B-deficient hCTLs (Figure 4A and Figure 6H) was decreased in the absence of IL-2. In summary, we found that loss of ARPC1B was accompanied by reduced TCR signaling, degranulation, granzyme B expression, and cytotoxicity in hCTLs. These results suggest that IL-2 regulates a plethora of signaling pathways that control gene transcription, protein synthesis, and the cytoskeleton (37), may provide partial compensation for the loss of effector function in ARPC1B-deficient hCTLs by upregulating the expression of an alternative ARPC1 isoform as well as granzyme B, a cytolytic protein required for in vivo antiviral immunity.

### ARPC1B deficiency impairs the polarization of cytotoxic machinery to the synapse.

As ARPC1B-deficient hCTLs displayed a marked killing defect associated with reduced TCR signaling and granule secretion, we asked whether the cytotoxic machinery polarized to the synapse in these cells. Quantitation of 3D images of fixed hCTL-target conjugates, stained for CD8 to identify hCTLs and the actin cytoskeleton (Supplemental Figure 2), showed that, while HD hCTLs were able to reorganize the actin cytoskeleton across the synapse (41%), showing either intermediate (22%) or ring-like phenotype (19%), only 10% of conjugates formed by ARPC1B-deficient hCTLs displayed an intermediate phenotype (Supplemental Figure 1, A and B, and Supplemental Figure 2A) and no conjugates formed a ring-like structure. Labeling conjugates with antibodies to CD8 and γ-tubulin allowed us to monitor centrosome polarization to the synapse. While 50% of HD hCTL conjugates polarized the centrosome to within 3 μm of the synapse, only 32% of ARPC1B-deficient hCTLs showed centrosome polarization to less than 3 μm of the synapse, demonstrating a reduction in the efficiency of centrosome polarization, potentially linked to reduced TCR signaling (Supplemental Figure 2B and ref. 38). Finally, we asked whether the secretory granules polarized to the synapse in the absence of ARPC1B by staining tubulin to visualize microtubules and by using antibody to LAMP1 to label the secretory granules (Supplemental Figure 2C). hCTLs were distinguished from target cells by the human-specific LAMP1 antibody, which does not recognize mouse target cells. No obvious differences were noted in the microtubule cytoskeleton of HD and ARPC1B-deficient hCTLs. However, polarization of the granules was impaired in ARPC1B-deficient hCTLs, with less than 10% of hCTLs showing granule polarization, while more than 40% of HD hCTLs showed polarized granules forming a bright cluster at the contact site between hCTLs and target cells (Supplemental Figure 2C). Hence, although ARPC1B-deficient hCTLs were able to secrete granules, not all granules clustered at the synapse. Thus, loss of ARPC1B not only disrupts actin reorganization across the synapse, but also impairs the polarization of the centrosome and the cytotoxic machinery to the synapse, weakening the overall effector function of CTLs.

### Reduced cell surface TCR-αβ, CD8, and GLUT1 in ARPCB1B-deficient hCTLs.

**Figure 7:**
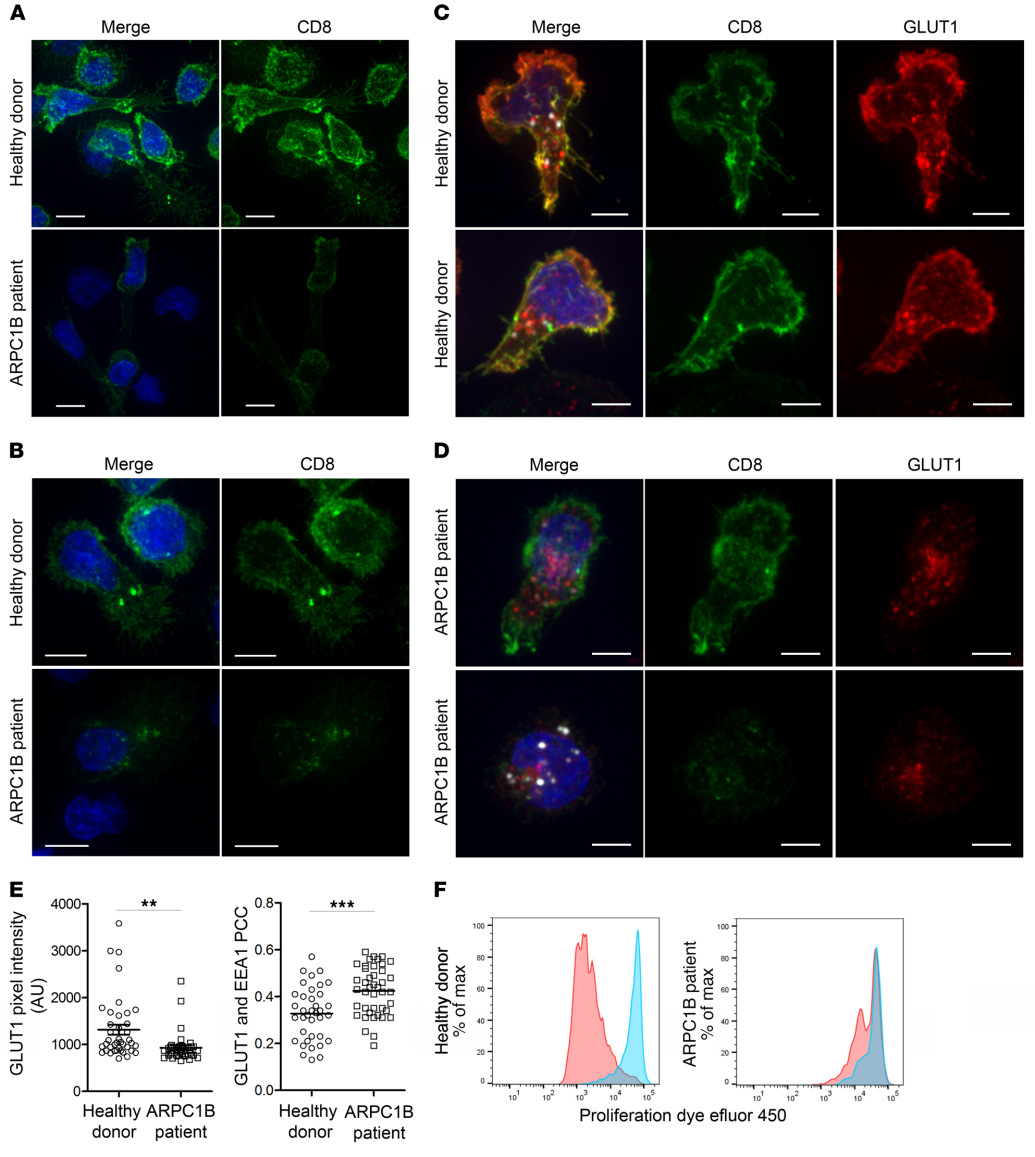
Absence of ARPC1B alters surface expression of CD8 and GLUT1 in hCTLs. (A–D) HD and ARPC1B-deficient patient hCTLs were fixed in PFA for 20 minutes, permeabilized, and stained with an antibody against CD8 alone (green) (**A** and **B**) or in combination with anti-GLUT1 (red) and anti-EEA1 (white) antibodies (**C** and **D**). Images are 3D reconstructions of *Z*-stack. Scale bars: 4 μm. (**E**) Measurement of the mean intensity of GLUT1 expressed in AU and the degree of colocalization with EEA1 expressed as PCC (see Methods) in HD and ARPC1B-deficient patient hCTLs based on images as sampled in **C** and **D**. HD, *n* = 41 cells; ARPC1B-deficient patient, *n* = 38 cells. *P* < 0.005 (unpaired *t* test). **P < 0.0013; ***P < 0.0002. (**F**) Flow cytometry analysis of the proliferation capacity of HD and ARPC1B-deficient patient hCTLs (gated on live CD8^+^ cells) in the absence (blue) or presence (red) of plate-bound anti-CD3 stimulation (1 μg/mL). All data are representative of 3 independent experiments.

In order to culture hCTLs from HD and the ARPC1B-deficient patient, we purified CD8^+^ T cells using negative selection, depleting with antibodies against markers for CD4^+^ T cells, B cells, NK cells, monocytes, granulocytes, and dendritic cells (see Methods). While purified HD T cells were 99% CD8 positive 13 days after stimulation, only 17% of the purified ARPC1B-deficient T cells expressed cell surface CD8 detected by flow cytometry, with 79% of the T cells in the population expressing neither CD8 nor CD4 (Supplemental Figure 3A). The median fluorescence intensities (MFI) for CD3 and CD8 were comparable in patient and HD, indicating that the number of CD8 cells in the population was specifically reduced (Supplemental Figure 3B). When we examined TCR-αβ expression, we also noticed a marked reduction in the number of ARPC1B-deficient cells expressing both TCR-αβ and CD8 compared with HD cells, 13% and 96%, respectively (Supplemental Figure 3C). However, the MFI of TCR-αβ staining in the ARPC1B-deficient cells that retained TCR-αβ and CD8 expression was not significantly reduced relative to that of their HD counterparts (Supplemental Figure 3D). No expression of TCR-γδ receptor was seen (data not shown). Immunofluorescence microscopy analysis showed CD8 was readily detected on the plasma membrane of HD cells, but only weakly on the plasma membrane of ARPC1B-deficient cells (Figure 7A). However, we noted that up to 10% of ARPC1B-deficient patient cells with no apparent CD8 on the plasma membrane showed intracellular puncta of CD8 (Figure 7B); furthermore, we observed perforin expression within cells lacking plasma membrane CD8 (Supplemental Figure 3F). These results suggested that these cells were CTLs that no longer expressed cell surface CD8. Therefore, we asked whether receptor recycling might be impaired in ARPC1B-deficient hCTLs.

Although Arp2/3 is perhaps best known for its role in modeling the cortical actin cytoskeleton, it has also been shown to have a role in the regulation of endocytosis and receptor recycling via the retromer and WASH complexes (39, 40). Therefore, we asked whether these pathways were affected in ARPC1B-deficient hCTLs. Labeling with antibodies against the retromer subunit VPS35 as well as FAM21, the subunit of the NPF WASH complex that interacts with the retromer (41–43), showed the proteins colocalized on intracellular vesicles in both HD and ARPC1B–deficient hCTLs and demonstrated that the retromer-dependent recycling machinery was not only present, but also correctly localized in both (Supplemental Figure 3G). Next, we asked whether the localization of GLUT1, a well-established cargo protein of the retromer recycling pathway (44, 45), was affected in ARPC1B-deficient hCTLs. We found that, although GLUT1 could be seen both on the plasma membrane and intracellularly in HD hCTLs, GLUT1 was not detected on the plasma membrane of ARPC1B-deficient hCTLs (Figure 7, C and D), resulting in a reduced GLUT1 intensity in ARPC1B-deficient hCTLs compared with HD (Figure 7, C–E). GLUT1 was only observed as an intracellular pool colocalizing with the endosomal marker EEA1 in ARPC1B-deficient hCTLs (Figure 7, D and E). We were also able to reproduce these findings in a second ARPC1B-deficient patient (Supplemental Figure 4). These results demonstrate that ARPC1B is required for retromer and WASH complex–mediated recycling, as GLUT1 is lost from the plasma membrane in ARPC1B-deficient hCTLs. Furthermore, our results suggest that TCR-αβ and CD8 also require retromer and WASH complex–mediated recycling to maintain cell surface levels in CTLs.

As GLUT1 is the major glucose transporter for hCTLs, required for cell metabolism and proliferation (46), we asked whether cell proliferation was affected by analyzing division of hCTLs over 5 days. While the majority of HD hCTLs underwent multiple divisions following stimulation with plate-bound anti-CD3, fewer ARPC1B-deficient hCTLs divided less frequently, revealing a defect in proliferation of ARPC1B-deficient hCTLs compared with HD (Figure 7F). We followed the fate of hCTLs derived from the ARPC1B-deficient patient after successive stimulations (see Methods and Supplemental Figure 5A). Although there was a comparable CD4/CD8 ratio in patient and HD PBMC initially, we observed a progressive decrease in the ARPC1B-deficient CD8^+^ population with successive stimulations (Supplemental Figure 5B). Stimulation of a CD8-purified population triggered a greater decrease in the percentage of CD8^+^ T cells in a ARPC1B-deficient patient compared with HD (Supplemental Figure 5, D and E). These results demonstrate the importance of ARPC1B in CTLs upon selection and proliferation. When ARPC1B was absent, GLUT1, the main glucose transporter in CTLs (47), together with TCR-αβ and CD8, was lost from the plasma membrane with profound consequences for CD8 CTL maintenance.

## Discussion

T and NK cell activation lead to a rapid reorganization of F-actin across the synapse, coordinating receptor signaling platforms and intracellular effectors to deliver an appropriate immune response (2, 48–50). Recent studies have begun to probe the role of the Arp2/3 complex in actin nucleation in T cells (51–53). The ARPC1 subunit is thought to play a regulatory role within the Arp2/3 complex, forming an interface together with ARPC5 and Arp2 that is critical for actin nucleation (54–56). Although deletions of many subunits of the Arp2/3 complex are embryonic lethal (57), deletion of ARPC1B is not, as it is predominantly expressed in the hemopoietic lineage (18), where ARPC1A expression is minimal. Patients lacking ARPC1B show combined immunodeficiency arising from loss of ARPC1B in different hemopoietic cells (18–22). Clinical presentations reported for these patients include platelet defects, mild thrombocytopenia, recurrent viral and bacterial infections, eczema, and enterocolitis, with different combinations of these defects varying among patients. Studies on ARPC1B-deficient T cells have described reduced proliferation in response to anti-CD3 and variable or reduced CD8^+^ T cell counts in the blood (21, 22). Furthermore, defects in actin accumulation at the synapse and during T cell migration have also been described (21). However, the molecular basis of the decreased proliferation and reduced CD8 population have remained elusive and whether CD8-mediated cytotoxicity was affected by loss of ARPC1B is not known.

Here, we show that absence of ARPC1B leads to a loss of CTL/CD8 cytotoxicity, with the defect arising at 2 different levels. First, ARPC1B is essential for the polymerization of actin in lamellipodia, required for cell motility and synapse formation. However, in addition, activation induces a decrease in the CD8^+^ cell population arising from the role of ARPC1B in retromer and WASH complex–mediated recycling of key surface receptors required for T cell activation (TCR and CD8) and proliferation (GLUT1).

We used 2 different approaches to examine the role of Arp2/3 in CTL-mediated cytotoxicity. First, using drug treatment on OT-I CTLs, we found that inhibition of Arp2/3 acted at several stages during CTL killing: we found that lamellipodia formation and the speed of migration were both inhibited upon CK666 treatment, as was the reorganization of actin across the synapse and the polarization of the centrosome that directs granules and the lethal hit. Cytotoxicity was reduced, with a modest reduction in degranulation, while TCR-mediated p-ERK activation was unaffected. Thus, the decreased target cell killing observed in these short-term cytotoxicity assays (1.5 hours) was most likely linked to defective lamellipodia formation during migration and subsequent synapse maintenance rather than a substantial signaling defect, consistent with recently published observations (58). Second, we observed a cytotoxicity defect in patient-derived ARPC1B-deficient hCTLs. However, in addition to impaired lamellipodia formation, substantial decreases in TCR signaling and degranulation were also observed, suggestive of a more profound role for Arp2/3 in CTLs beyond those observed in the short-term inhibitor assay.

In the absence of ARPC1B, we saw an upregulation of the nonhematopoietic isoform of ARPC1A, which was enhanced in the presence of IL-2. However, upregulation of ARPC1A did not prevent the loss of cytotoxicity observed in the ARPC1B-deficient patient, suggesting only weak compensation by ARPC1A or a nonredundant role of ARPC1B in CTLs. Similarly, a lack of complementation has been observed in ARPC1B–deficient platelets with upregulated ARPC1A (19). A defect in NK cell degranulation in ARPC1B-deficient patients has been reported, with IL-2 increasing degranulation in both control and patient (22). In contrast to Arp2/3 inhibitor assays on OT-I CTLs, purified CD8 cells derived from the ARPC1B-deficient patient showed reduced TCR signaling together with reduced degranulation and cytotoxicity compared with the HD population. Starting with purified CD8 cells, after TCR stimulation, which is required to maintain hCTL population in vitro, we found a reduction in patient cells expressing both TCR-αβ and CD8. Loss of TCR-αβ and CD8 on the plasma membrane in ARPC1B-deficient hCTLs could account for the decreased TCR activation–induced p-ERK signaling and contribute to the reduced cytotoxicity that we observed.

It is well documented that activation of the TCR leads to its rapid internalization and recycling back to the plasma membrane is required for maintaining surface levels of TCR (59–62). We noted that CD8 could be seen within ARPC1B-deficient cells, but not on the plasma membrane, suggesting that CD8 follows the same fate as TCR. Furthermore, patient cells that had lost CD8 were found to express the cytolytic protein perforin, confirming that these cells were CTLs. Therefore, Arp2/3 is essential for the maintenance of CD8 and TCR on the cell surface. Arp2/3 plays a key role in generating actin patches on endosomes that facilitate the transport of selected transmembrane proteins back to the plasma membrane via the retromer and WASH complex recycling pathway (13, 15, 63). We found that GLUT1, a well-characterized cargo protein of the retromer/WASH pathway (15, 44, 64, 65), was also lost from the plasma membrane in ARPC1B-deficient hCTLs; this was not associated with abnormal endosomes morphology as observed in the absence of WASH complex (66). Moreover, ARPC1B-deficient patients do not exhibit symptoms of spastic paraplegia or Parkinson’s disease, due to mutation in the strumpellin subunit of the WASH complex and mutation in the VPS35 subunit of the retromer complex, respectively (67–69). Therefore, the loss of ARPC1B does not affect the retromer and WASH machinery per se, but rather the F-actin–dependant endosome–to–cell surface recycling that relies on Arp2/3 activity (70). As GLUT1 is a key nutrient transporter required for T cell activation (71, 72), proliferation (46), and effector functions (73, 74), loss of GLUT1 could account for the impaired proliferation we observed in our experiments. We propose that loss of surface GLUT1 would likely contribute to the CD8 lymphopenia in the ARPC1B-deficient patient. Our results emphasize the importance of retromer-mediated recycling in the maintenance of TCR, CD8, and GLUT1 on the cell surface of CTLs.

Based on our findings, we propose that ARPC1B deficiency results in (a) the loss of lamellipodial contact, which is pivotal for synapse maintenance and CTL secretion; and (b) a reduction of TCR, CD8, and GLUT1 on the plasma membrane; these 3 receptors are essential for TCR activation, effector function, and proliferation in CTLs. As receptor recycling is triggered upon TCR activation (62, 75), the loss of these 3 receptors is exacerbated upon (re)activation. Thus, each repeated challenge will result in a relative decrease in the specific CTL/CD8 population and a reduced ability to respond, driving the susceptibility to viral disease (22). The lack of properly induced and sustained CD8^+^ T cell activity in ARPC1B deficiency may underlie the severe acute and prolonged viral infections observed in many of the patients, being either systemic (CMV and EBV infections) or localized (including respiratory syncytial virus [RSV] bronchiolitis, and adenovirus pneumonitis as well as HPV and molluscum contagiosa virus [MCV] skin infections) (22).

In conclusion, we find that the ARPC1B subunit of the Arp2/3 complex is essential for CTL cytotoxicity, acting not only by regulating actin reorganization at the synapse, but also by controlling the cell surface levels of TCR, CD8, and GLUT1 via its role in retromer and WASH-mediated recycling. Our study indicates that mutations in *ARPC1B* will trigger progressive CTL activation–induced immunodeficiency and provides mechanistic insights into the immune dysregulation observed in this disease.

## Methods

### Antibodies and reagents.

The following primary antibodies were used: rat anti-mouse CD8 (clone YTS105, gift from Herman Waldmann, University of Oxford, Oxford, United Kingdom), rabbit anti-human LAMP1 (AS120) (76), mouse anti-tubulin (TAT-1, gift of Keith Gull, University of Oxford, Oxford, United Kingdom), mouse anti-γTubulin (clone GTU-88, T6557, MilliporeSigma), rabbit anti-γTubulin (T5192, MilliporeSigma), mouse anti–β-actin (clone AC-15, A5441, MilliporeSigma), mouse anti-actin (clone AC-40, catalog A3853, MilliporeSigma), rabbit anti–F-actin (catalog A2066, MilliporeSigma), rabbit anti-ARPC2 (catalog 07-227, MilliporeSigma), rabbit anti–p-ERK1/2 (clone D13.14.4E, 4370S, Cell Signaling), mouse anti-ERK1/2 (clone L34F12, 4696S, Cell Signaling), rabbit anti-ARPC1B (catalog HPA004832, MilliporeSigma), rabbit anti-ARPC1A (catalog HPA004334, MilliporeSigma), rabbit anti-calnexin (catalog C4731, MilliporeSigma), mouse anti–granzyme B (clone 2C5/F5, catalog 550558, BD Biosciences — Pharmingen), rabbit anti-GLUT1 (catalog ab15309, Abcam), mouse anti-VPS35 (clone B5, catalog SC374372, Santa Cruz Biotechnology Inc.), rabbit anti-FAM21 (catalog ABT79, EMD Millipore), mouse anti-EEA1 (clone 14-EEA1, catalog 610456, BD Biosciences), and mouse anti-human CD8α–Alexa Fluor 488 (clone 37006, catalog FAB 1509G, R&D Systems). The following secondary antibodies from Thermo Fisher were used: Alexa Fluor 488 goat anti-mouse IgG (catalog A11029), Alexa Fluor 488 goat anti-rat IgG (catalog A11006), Alexa Fluor 555 goat anti-rabbit IgG (catalog A21429), Alexa Fluor 647 goat anti-mouse IgG (catalog A21236), Alexa Fluor 633 goat anti-rabbit (catalog A21071). Additional reagents used were as follows: phalloidin Alexa Fluor 555 (catalog A34055, Thermo Fisher) or Alexa Fluor 647 (catalog A22287, Thermo Fisher), CK666 (catalog ab1412131, Abcam), and CK689 (182517, catalog EMD Millipore).

### Primary cell culture.

Spleens collected from C57BL/6 OT-I Rag1^-/-^ mice were homogenized through a 70 μm filter. Splenocytes from OT-I mice were stimulated with 10 nM of OVA_257–264_ [SIINFEKL] peptide (AnaSpec) and cultured for 3 days to generate OT-I CTLs. OT-I CTLs were maintained for 8 to 10 days in mouse T cell media consisting of RPMI 1640 medium (Thermo Fisher) supplemented with 10% (v/v) FBS (Labtech), ≥100 U/mL recombinant murine IL-2 (212-12, PreproTech), 50 μM β-mercaptoethanol (Thermo Fisher), 100 U/ml penicillin-streptomycin (MilliporeSigma), 2 mM l-glutamine (MilliporeSigma), and 1 mM sodium pyruvate (Thermo Fisher).

PHA blasts from frozen human PBMCs isolated from HD or ARPC1B-deficient patients carrying a homozygous mutation, c.491_495delinsCCTGCCC on exon 7 (18) or mutation c.897_910delCGAGCGCTTCCAGA on exon 10 (22), were stimulated with 10^6^ cells/mL of γ-irradiated (30 Gy using a Gammacell 1000 Irradiator) PBMCs and 1 μg/mL PHA (MilliporeSigma) and grown in human T cell media composed of the following: RPMI 1640 supplemented with 5% (v/v) human serum (H6914, MilliporeSigma), 2% (v/v) recombinant IL-2 (≥100U/mL; produced from IL-2–expressing X63 myeloma cells), 50 μM β-mercaptoethanol, 0.075% sodium bicarbonate (Thermo Fisher), 2mM l-glutamine, and 1 mM sodium pyruvate. CD8^+^ cells were purified by negative selection at day 10 after stimulation using the Dynabeads Untouched Human CD8 T cells Isolation Kit, depleting CD4, CD14, CD16a, CD16b, CD19, CD36, CD56, CD123, and CD235a (Thermo Fisher). hCTLs were restimulated every 3 weeks with irradiated PBMCs and 1 μg/mL PHA as described earlier.

### Cell line culture.

To generate P815-NucLight Red, P815 was transduced with the IncuCyte NucLight Red Lentivirus (4625, Essen Bioscience) to stably label the nuclei with mKate2 red fluorescent protein. After 3 days, successfully transduced cells were sorted by FACS (Becton Dickinson Influx Cell Sorter). Subsequently, P815-NucLight Red cells were kept under continuous selection conditions with media containing 1 μg/mL puromycin (Thermo Fisher). HEK293T, EL4, EL4-Farnesyl-5-TagBFP2 (blue EL4) (5), P815-FARN-TagBFP2 (blue P815), and P815 target cells were maintained in DMEM medium (Thermo Fisher) containing 10% FBS and 100 U/mL of penicillin-streptomycin.

### Cloning and expression of lentiviral mApple-Lifeact and ARPC3 nucleofection.

The plasmid mApple-Lifeact-7, a gift from Michael Davidson (Florida State University, Tallahassee, Florida, USA) (Addgene plasmid 54747), was subcloned into the XhoI and NotI restriction sites of the pHR-SIN lentiviral vector. For virus production, 6 μg pHR-SIN-mApple-Lifeact-7 was transfected with 4 μg pMDG VSV-G (envelope plasmid) and 4 μg pCMV delta 8.9 (packaging plasmid) into HEK293T cells (at 70% confluency) using TransIT transfection reagent (Mirrus) and cultured overnight. Supernatant was collected after 48 hours, centrifuged, and filtered through a 0.45 μm filter before concentration with LentiX concentrating solution (Clontech, Takara). 10^7^ CTLs were transduced with concentrated viral particles in the presence of 6 μg protamine sulfate (P4020-1G, MilliporeSigma) in 1 mL of T cell media and left for 4 hours at 37°C. CTLs were diluted to 10^6^ cells/mL before stimulation at a 1:1 ratio with irradiated PBMCs in the presence of 1 μg/mL PHA.

To generate the EGFP-ARPC3 construct, mRNA was extracted from OT-I CTLs using an RNEasy Mini Kit (QIAGEN). OT-I cDNA was generated from this using the AccuScript High-Fidelity First Strand cDNA Synthesis Kit (Agilent). To create a crippled CMV promoter, the majority of the enhancer region was deleted from EGFP-N1 (Clontech, Takara) using the restriction enzyme AatII (Thermo Fisher) to generate the plasmid delCMV-EGFP-N1 described in Watanabe and Mitchison (77). ARPC3 primers were used to amplify the coding region based on NCBI Reference Sequence NM_019824.4: forward, 5′-ATGCCGGCATACCACTCTTCTCTC-3′; and reverse, 5′-CTGCCCAGGCCCCGAAAGAC-3′ with XhoI and BamHI restriction sites added. The product was ligated into delCMV-EGFP-N1, and 5 μg of DNA was nucleofected with 5 μg mApple-Lifeact-7 using the P3 Primary Cell Kit (V4XP-3024, Lonza) following the manufacturer’s recommendation for the 4D nucleofector (Lonza).

### Cytotoxicity assay.

OT-I CTLs or hCTLs were resuspended in killing assay media (phenol red–free RPMI 1640 containing 2% FBS) and mixed at effector-to-target ratios shown with EL4 (pulsed with 1 μM OVA_257–264_ for 1 hour at 37°C) or P815 (mixed with 0.5 μg/mL mouse anti-human CD3 (clone UCHT1, catalog 555330, BD Pharmingen), respectively. For experiments with CK666 or CK689, OT-I CTLs were treated 10 minutes at 37°C with 90 μM of the drug, which was also added to the killing assay media. For experiments without IL-2, hCTLs were washed and cultured in media without IL-2 for 16 hours prior to the assay. After 1.5 hours to 2 hours incubation, the percentage of target cell lysis was determined using the CytoTox 96 Non-Radioactive Cytotoxicity Assay following the manufacturer’s instructions (Promega).

### IncuCyte killing assay.

Flat bottom 96-well plates (3595, Corning) were coated with 50 μL/well Poly-l-ornithine (PLO) (P4957, MilliporeSigma) for 1 hour at room temperature (RT) and subsequently dried for 1 hour at RT. P815-NucLight Red (2500 cells) were seeded per well in phenol red–free human T cell media without IL-2 and left to attach to the plate overnight at 37°C. Additionally, hCTLs (day 11 after stimulation) were cultured in human T cell media without IL-2 overnight at 37°C. The following day, hCTLs were added to the P815-NucLight Red target cells in the presence or absence of 0.5 μg/mL anti-CD3 antibody (clone UCHT1, catalog 555330, BD Biosciences — Pharmingen) at an effector-to-target ratio of 8:1. Loss of red fluorescence (i.e., targeted cell death) was monitored every 30 minutes for 8 hours using the IncuCyte S3 live-cell analysis system (Essen Bioscience) and plotted as percentage of lysis.

### Flow cytometry.

Degranulation assays were carried out as previously described (78) with the exception of hCTLs being washed and maintained in media without IL-2 (for 16 hours) where indicated. Briefly, OT-I CTLs (day 7 after stimulation) or hCTLs (day 12 after stimulation) were mixed at a 1:1 ratio with their respective target cells and incubated for 2 to 3 hours at 37°C in the presence of rat anti-mouse CD107a-PE (clone 1D-4B, catalog 12-1071-83, eBioscience) or mouse anti-human CD107a-PE (clone H4A3, catalog 328608, BioLegend) and in the case of hCTLs, anti-CD3 antibody (clone UCHT1, catalog 555330, BD Biosciences). Where indicated, CK689 or CK666 was added in the media at 90 μM. CTLs were stained with either rat anti-mouse CD8a-APC (clone 53-6.7, catalog 100712, BioLegend) or mouse anti-human CD8-APC (clone MEM-31, catalog 26004, Abcam). For surface staining of transmembrane receptors, hCTLs at day 13 after stimulation were washed in FACS buffer (D-PBS + 1% FBS), resuspended at 0.5 × 10^6^ cells/mL, and costained for 30 minutes at 4°C with mouse anti-human CD3-PE (clone UCHT1, catalog 12-0038-42, eBiosciences), mouse anti-human CD8-FITC (clone UCHT-4, catalog F-0772, MilliporeSigma), mouse anti-human CD4-APC (clone RPA-T4, catalog 17-0049-42, Thermo Fisher), or mouse anti-human TCR-αβ–APC (clone IP26, catalog 17-9986-42, eBioscience). The isotype control antibodies used were mouse IgG1-PE (catalog ab81200, Abcam), mouse IgG2A-FITC (catalog ab81197, Abcam), and mouse IgG1κ-APC (catalog 17-4714-82, eBioscience). Cells were washed, resuspended in FACS buffer, and acquired on a BD Accuri C6 Flow Cytometer (BD Biosciences). For analysis of CD4/CD8 ratios, samples were washed in PBS, resuspended at 10^6^ cells/mL, and costained with mouse anti-human BUV395 (clone G42-8, catalog 743070, BD Biosciences), mouse anti-human CD3-BV711 (clone SK7, catalog 344837, BioLegend), rat anti-human CD4-PE (clone A161A1, catalog 357404, BioLegend), and a Live/Dead fixable NIR (catalog 423105, Thermo Fisher) for 25 minutes on ice. Unbound antibodies were removed by washing cells in PBS, 1% FCS; samples were fixed in 4% PFA for 10 minutes and resuspended in PBS prior to acquisition on a LSR Fortessa Flow Cytometer (BD Biosciences). All samples were gated on forward and side scatter and for singlets, and 20,000 single cells (for surface staining assay), 100,000 single cells (for CD4:CD8 ratio experiment), or 10,000 CD8^+^ cells (for degranulation assay) were recorded. Analysis was performed using FlowJo 10 software.

### Proliferation assay.

Flat-bottom 96 well plates were prepared with or without anti-CD3 coating at 1 μg/mL (clone OKT3, catalog 14-0037-82, eBioscience). hCTLs were stained with 10 μM cell proliferation dye eFluor 450 (65-0842-85, Thermo Fisher) for 10 minutes in the dark at 37°C, followed by quenching with FBS-containing media for 5 minutes. Subsequently, hCTLs were washed 3× with FBS-containing media, and 20,000 hCTLs were seeded per well. After 5 days, cells were washed 2× in ice-cold D-PBS (Thermo Fisher) prior to staining with an anti–CD8-APC (clone SK1, catalog 344722, BioLegend) or anti-CD4-PE (clone A161A1, catalog 357404, BioLegend) and Live/Dead fixable yellow (catalog L34959, Thermo Fisher). hCTLs were washed in PBS, 1% FCS prior to analysis on a LSR Fortessa flow cytometer. Samples were gated on live CD8^+^ cells; results were analyzed using FlowJo 10 software.

### Immunoblotting.

CTLs were lysed in 50 mM Tris-HCl (pH 8), 150 mM NaCl, 1 mM MgCl_2_ (M2670, MilliporeSigma), 2% Triton X-100 (T8787, MilliporeSigma) buffer at a concentration of 2 × 10^7^ cells/mL. For phospho-immunoblot prior to lysis, OT-I CTLs were pretreated for 10 minutes at 37°C with 90 μM CK689 or CK666 in culture media and subsequently left untreated or stimulated with 50 nM of PMA (P8139, MilliporeSigma) or OVA_257–264_ (1 μM) for 15 minutes. hCTLs were stimulated for 15 minutes at 37°C with 0.5 μg/mL or 1 μg/mL plate-bound anti-CD3 (clone OKT3, catalog 14-0037-82, eBioscience) before lysis. CTLs were lysed in phospho-lysis buffer (100 mM NaCl, 20 mM Tris-HCL pH 7.4, 10% glycerol [G7893, MilliporeSigma], 1 mM EDTA, 5 mM NaF [S7920, MilliporeSigma], 2 mM sodium orthovanadate [S6508, MilliporeSigma], 0.2% Triton X-100) supplemented with PhosSTOP (04906845001, Roche) and Protease Inhibitor Cocktail (04693132001, Roche). Lysates were loaded in 4× NuPage sample buffer (NP0007, Thermo Fisher) on a 4%–12% NuPage Bis-Tris Gel (NP0336, Thermo Fisher) under reducing conditions in MES Buffer (NP0002, Thermo Fisher), transferred to nitrocellulose membrane (10600003, GE Healthcare), and incubated with the indicated primary antibodies followed by goat anti-rabbit IgG (A16096, Thermo Fisher) or goat anti-mouse IgG (32430, Thermo Fisher) secondary antibody conjugated to HRP. Chemiluminescence was detected with ECL Prime kit (RPN2236, GE Healthcare) and recorded on a Chemidoc Imaging System (Bio-Rad). Quantitation of band intensity was performed using Image Lab 4.1 software (Bio-Rad). See complete unedited blots in the supplemental material.

### Immunofluorescence and live cell imaging.

OT-I CTLs or hCTLs were mixed at a 1:1 ratio in serum-free RPMI with OVA-pulsed EL4 or P815 (mixed with 1μg/mL of human anti-CD3 (clone UCHT1, catalog Ab00112, Absolute Antibody), respectively, and incubated for 20 to 40 minutes at 37°C. Cells were fixed for 15 minutes in 4% paraformaldehyde (15710-S, Electron Microscopy Systems) or 5 minutes in ice-cold methanol (10674862, Thermo Fisher), permeabilized in 0.1% Triton X-100, and blocked in 2% BSA in PBS (40 minutes). Samples were labeled overnight at 4°C with primary antibodies followed by fluorophore-conjugated secondary antibodies (1 hour at RT). Nuclei were stained for 5 minutes at RT with Hoechst 33342 (H3570, Thermo Fisher), and samples were mounted in ProLong Diamond Antifade Reagent (P36961, Thermo Fisher). *Z*-stack series of 0.4 μm intervals were taken with the ×60 oil objective (numerical aperture 1.42) or ×100 oil objective (numerical aperture 1.45).

For live cell imaging, 24 hours prior to imaging, OT-I CTLs were transfected with 2.5 μg of Lifeact-EGFP alone or with 2.5 μg of PACT-mRFP (79) using Mouse T cell Nucleofector Kit (Lonza). Blue EL4 target cells were pulsed with 1 μM OVA peptide for 1 hour at 37°C and washed twice in serum-free DMEM. For hCTLs, blue P815 target cells were washed twice in serum-free DMEM and incubated with anti-CD3 antibody for 10 minutes at 37°C. Targets were plated on 35 mm glass-bottom culture dishes (P36G-1.5-14-C, MatTek) coated with 0.5 μg/mL ICAM-1/FC (796-IC-050, R&D Systems). Approximately 2 × 10^6^ OT-I CTLs or hCTLs expressing fluorescent constructs in T cell media with 25 mM HEPES (15630080, Thermo Fisher) were added dropwise to plated targets. For CK666 treatment, drug was added onto the dishes and kept during imaging. Interactions were imaged using a ×60 silicone objective (numerical aperture 1.3). *Z*-stacks with 0.8 μm intervals were imaged every 12 seconds. To measure speed of cell migration, EGFP-Lifeact expressing OT-I CTLs were pretreated for 10 minutes at 37°C with 90 μM CK666 or CK689 and drugs were kept during imaging. For each condition, images were taken every 10 seconds for 10 minutes with ×20 objective (numerical aperture 0.75). To observe OT-I CTL migration behavior, *Z*-stacks with 1 μm intervals were taken at higher frequency (every 3 seconds for 5 minutes) using the ×60 silicone objective.

Images were obtained on an IX81 Olympus microscope equipped with an Andor Revolution system fitted with a CSU-X1 spinning-disk unit (Yogogawa), a 1024 × 1024 iXon Ultra EMCCD camera, a 2× camera adaptor (Andor Technology, Oxford Instruments), an environmental chamber maintained at 37°C and 5% CO_2_ (Okolabs), and lasers exciting at 405 nm, 488 nm, 561 nm, and 633nm. All analyses were performed using Imaris software (Bitplane).

### Super-resolution structured illumination microscopy.

For super-resolution structured illumination microscopy (SR-SIM), coverslips (474030-9000-000, Zeiss) cleaned as previously described (80) were coated with 0.01% poly-l-lysine (MilliporeSigma) for 15 minutes, washed with PBS, and coated with 10 μg/mL hamster humanized anti-CD3 antibody (ChAgly, a gift from Herman Waldmann) or 0.5 μg/mL ICAM-1 (796-IC-050, R&D) for 2 hours at 37°C. Coverslips were washed in D-PBS and equilibrated for 30 minutes with phenol red–free T cell media prior to addition of hCTLs (2 × 10^6^ cells/mL). Cells were allowed to adhere for 12 minutes and subsequently fixed and stained as described in the *Immunofluorescence and live cell imaging* section. Samples mounted in ProLong Gold (P36930, Thermo Fisher) were cured (48 hours at RT) and examined on a Zeiss ELYRA microscope (Cambridge), using a ×63 Plan Apochromat oil immersion objective (numerical aperture 1.4) and 405 nm, 488 nm, and 561 nm laser lines. Images were processed using Zen software (Zeiss) and visualized in Imaris.

### Electron microscopy.

P815 targets were washed and resuspended in serum-free media at 1 × 10^6^ cells/mL. hCTLs at day 12 after stimulation were preincubated for 5 hours with HRP (Boehringer Ingelheim) at 1 mg/mL directly added to medium to load endocytic compartments, then washed, resuspended at 1 × 10^6^ cells/mL in serum-free media, mixed 1:1 with the P815 targets in the presence of 1 μg/mL mouse anti-human CD3 antibody (clone UCHT1), and left 3 to 5 minutes in suspension before plating in 4-well Nunc plates and incubating at 37°C for 25 minutes to allow cell/cell interactions. Samples were fixed in 1.5% glutaraldehyde/2% paraformaldehyde, then washed and processed for DAB cytochemistry and EPON embedding as described previously (81). Samples were viewed on a FEI Tecnai G2 Spirit BioTWIN transmission EM (Eindhoven) and images captured using a Gatan 4K US1000 CCD camera and FEI TIA software. Images were viewed in Adobe Photoshop CS6.

### Image analysis and quantitation using IMARIS.

For speed of migration measurement, the Spot detection module (size = 15 μm) was used to track moving cells. Nonadherent (floating) cells were excluded and 5–11 cells were selected and subjected to the analysis tool to calculate their respective speed of migration.

With the series of confocal *Z*-stack acquired, CD8 staining was used to distinguish CTLs using the Surface tool, source channel: channel 2 = 488 with a surface detail of 0.5 μm. After threshold adjustment and background elimination, a mask was applied to channel 561 and channel 640 of CD8^+^ cells, prior to analysis using the colocalization tool. Results were expressed as a PCC where values between ± 0.50 and ± 1 mean a high degree of colocalization, values between 0.30 and ± 0.49 a moderate degree, and values below ± 0.29 a low degree. For intensity measurements, CD8^+^ cells identified by the surface tool were submitted to the analysis tool module, which computes the intensity mean in the third channel (corresponding to the 561 channel). Values were then plotted in GraphPad Prism.

For actin depletion measurements, the ×60 objective was positioned in the middle of the slide and 10 fields of view were successively acquired from the left to the right end. The oblique slicer tool (with a depth of 1.23 μm) was applied at the interface between the CTLs contacting a target to generate the en face view of the synapse. A snapshot of the synapse was taken and the intensity of actin quantified using ImageJ (NIH) as shown in Supplemental Figure 1. All CD8-positive cells and conjugates present in the field of view were identified and counted manually.

### Statistics.

GraphPad Prism was used to create all graphics and for statistical analysis. A 2-tailed Student’s *t* test was used where indicated to compare mean values. P < 0.05 was considered significant.

### Study approval.

This research was regulated under the Animals (Scientific Procedures) Act 1986 Amendment Regulations 2012 following ethical review by the University of Cambridge Animal Welfare and Ethical Review Body (AWERB). Heparinized venous blood was collected from HDs and ARPC1B patient (18) after informed consent had been obtained. The study was approved by the Academic Medical Center Institutional Medical Ethics Committee in accordance with the 1964 Declaration of Helsinki.

## Author contributions

LOR, TWK, MNJS, and GMG designed the study. LOR and GMG wrote the manuscript. LOR generated and analyzed data shown in Figures 1–7 and Supplemental Figures 1–4; KS generated data shown in Figure 6C, Figure 7E, and Supplemental Figure 4, D and E; MJ and LOR generated data shown in Figure 1A; YA and MJ generated data shown in Figure 3D and Supplemental Videos 3 and 4; YA generated data shown in Figure 3C and Supplemental Videos 1 and 2; JCS generated data shown in Figure 5A; CMGB generated the EGFP-ARPC3 construct used in Figure 1C.

## Supplementary Material

Supplemental data

Supplemental Video 1

Supplemental Video 2

Supplemental Video 3

Supplemental Video 4

Supplemental Video 5

Supplemental Video 6

## Version 1 11/11/2019

Electronic publication

## Version 2 12/02/2019

Print issue publication
