# Supplementary material for: Loss of ARPC1B impairs cytotoxic T lymphocyte maintenance and cytolytic activity
Source: J Clin Invest. 2019 Nov 11;129(12):5600–14. doi: 10.1172/JCI129388 (PMC6877333; doi:10.1172/JCI129388)
Supplement: Supplemental data [file jci-129-129388-s334.pdf]

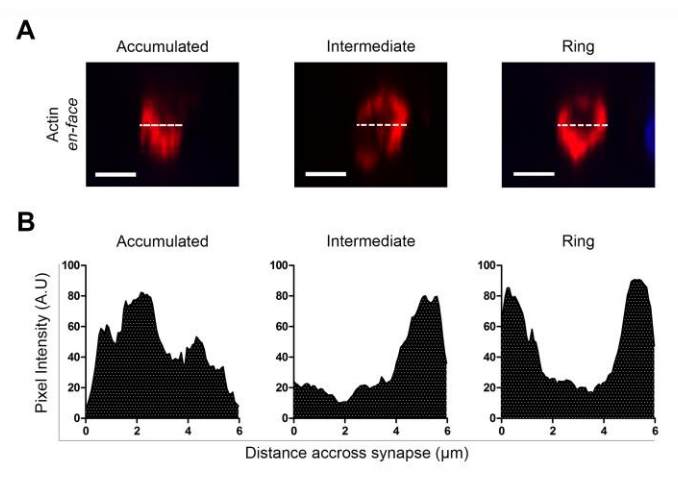

Supplemental Figure 1: Determination of actin phenotype across the synapse. (A) *En-face view* from 3D reconstruction of z-stack taken from fixed images that were used to generate the quantitative graph in Figure 3B, exemplifying the actin reorganization across the synapse. Images from OT-I-EL4 conjugates treated with CK689. White line represents the distance across the synapse plotted in graphs in B. Scale bar: 5µm. (B) Intensity plots of actin staining obtained from the line drawn in (A) for the 3 different actin phenotypes. *Accumulated*: actin across the synapse. *Intermediate*: uneven depletion of actin with intensity dropping at the centre of the synapse. *Ring*: centrally depleted actin with pixel intensity <30% left at the centre (within 2-4 microns) and >70% increase at the perimeter.

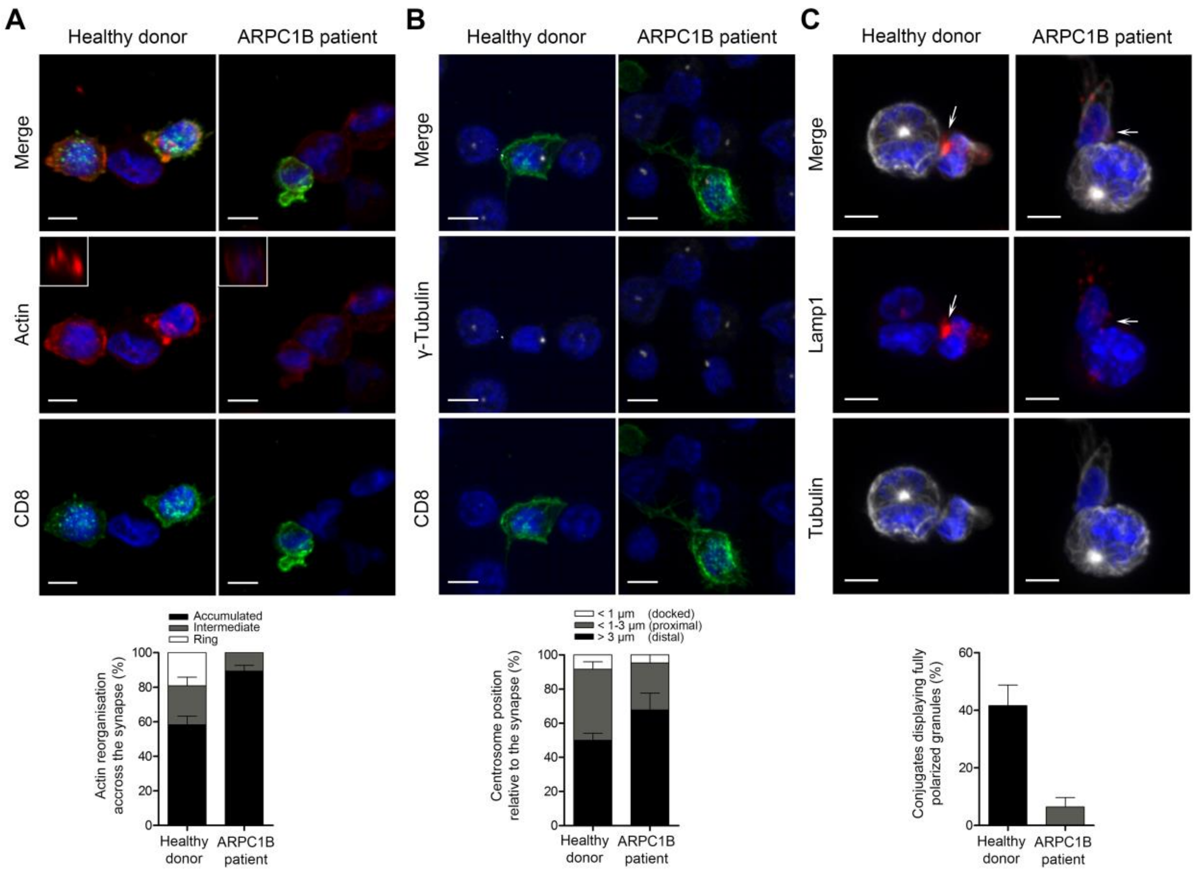

Supplemental Figure 2: Impaired actin reorganization and polarization of the cytotoxic machinery in ARPC1B-deficient hCTL. HD or ARPC1B-deficient patient hCTL conjugated to P815 for 35min, fixed and co-stained with CD8 (green) and actin (red) (A) or CD8 (green) and γ-Tubulin (white) (B) or LAMP1 (red) and α-Tubulin (white) (C). White box shows *en-face* view of the actin across the synapse. 3D reconstructions of z-stack are shown and used to quantitate the actin phenotype (A, bottom graph), the distance of centrosome relative to the synapse (B, bottom graph) and the presence or absence of LAMP1 cluster (white arrow) at the synapse (C, bottom graph). Scale bars: 5μm. Data are mean of 3 independent experiments. Error bars are SEM.

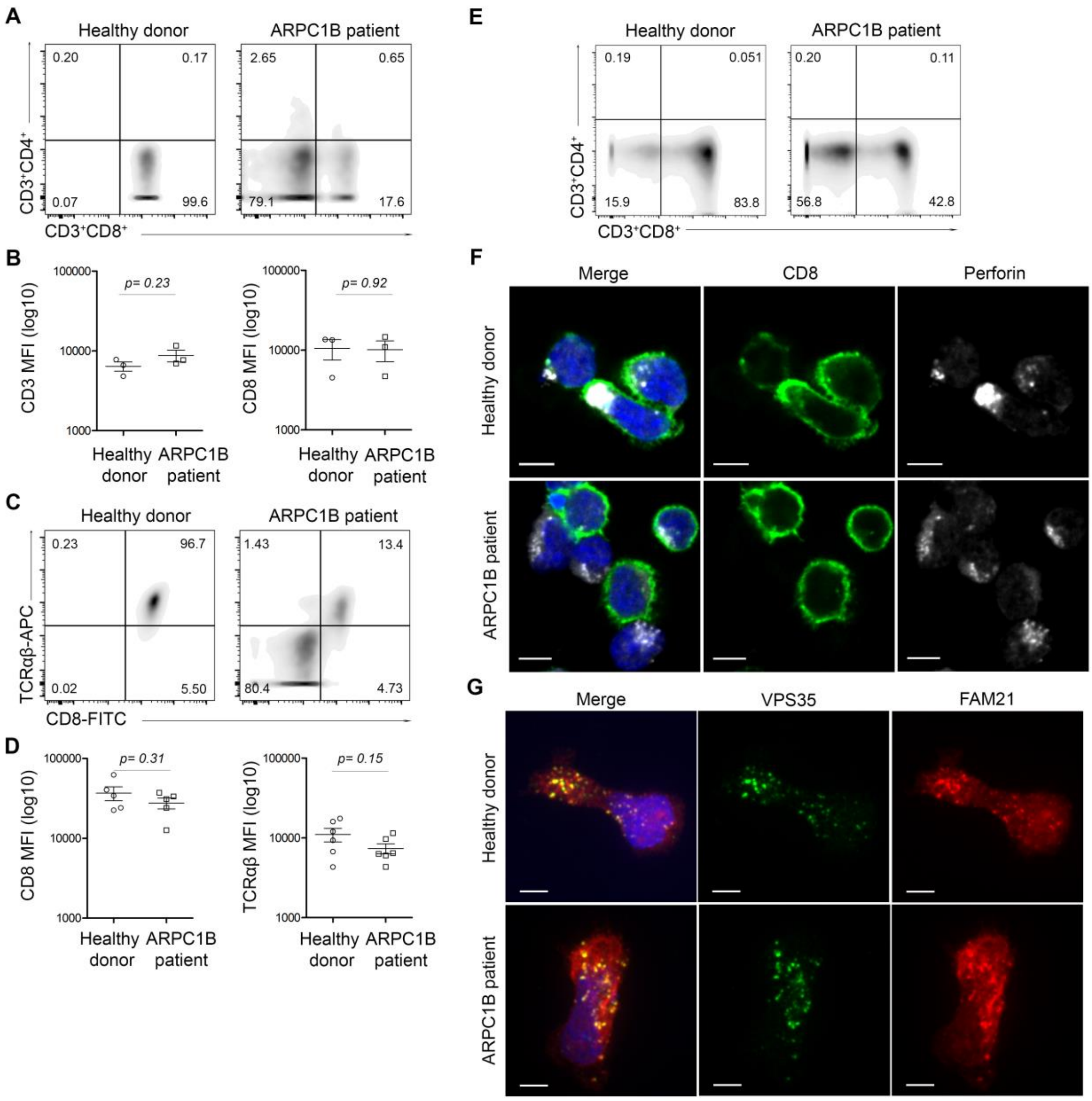

Supplemental Figure 3: Surface expression of CD8 and TCRαβ and localization of retromer recycling machinery in ARPC1B deficient hCTL. (A-D) Flow cytometry analysis of purified HD and ARPC1B-deficient patient CTL at day 13 post stimulation. (A) CD4:CD8 ratio within the $CD3^+$ population (data representative of 3 independent experiments). (B) Quantitation derived from $CD3^+CD8^+$ double-positive population as shown in (A). (C) Surface expression of CD8 and TCRαβ within population gated on single cells. Numbers in quadrants indicate percent cells in each. (D) Quantitation derived from $CD8^+TCR\alpha\beta^+$ double positive population as shown in (C); data are mean of 5 experiments, error bars are SEM; *p value > 0.05* (unpaired *t*-test). (E) Flow cytometry analysis of the CD4:CD8 ratio within the $CD3^+$ population of purified HD and ARPC1B-deficient hCTL. (F) Purified HD and ARPC1B-deficient hCTL from (E) were

fixed in PFA, permeabilized and co-stained with CD8 (green) and perforin (white). Scale bars: 5µm. (G) HD and ARPC1B-deficient hCTL were co-stained with VPS35 (green) and FAM21 (red). Scale bars: 3µm. Data are representative of 3 independent experiments except (E) and (F) (n=1).

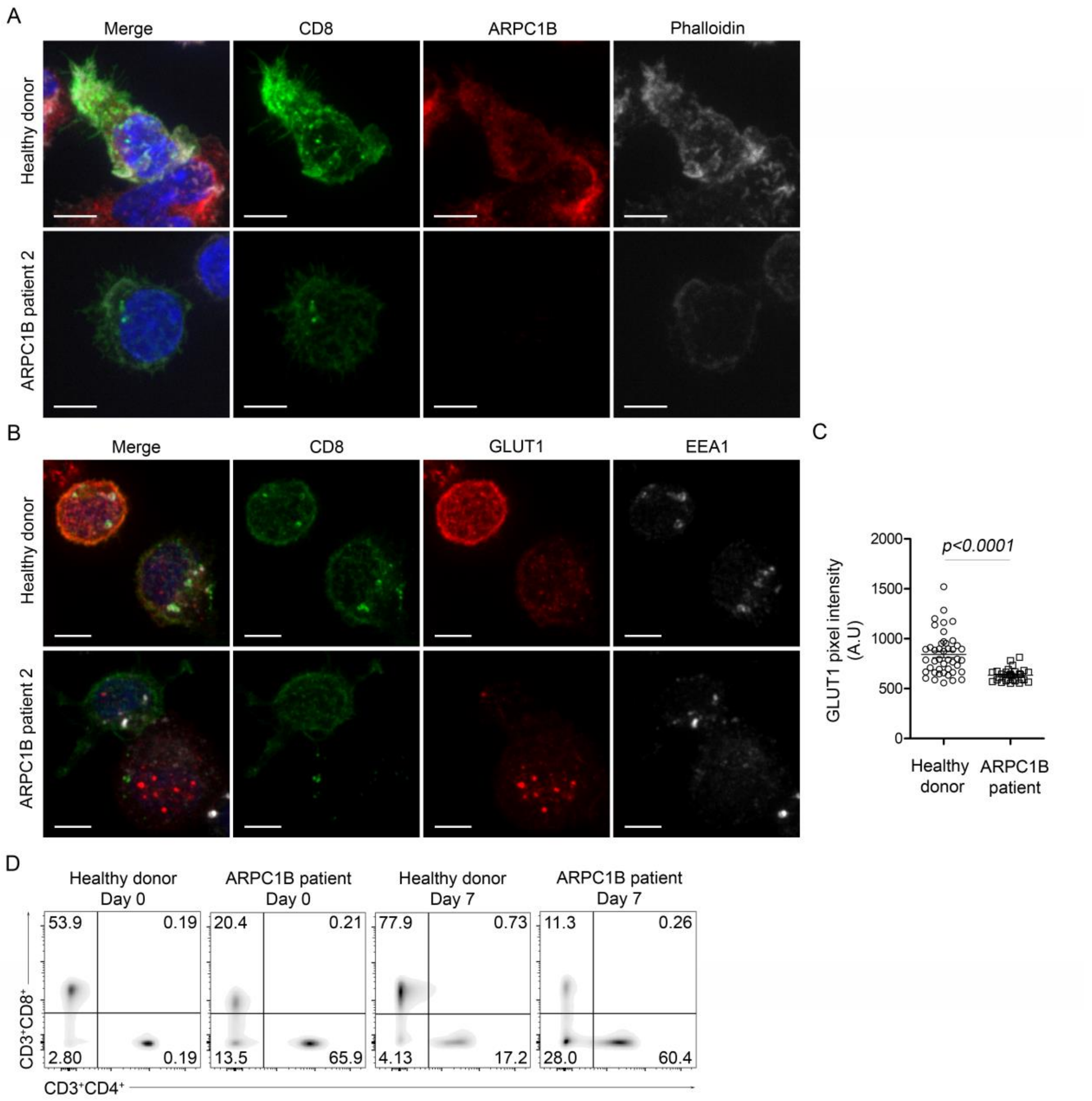

Supplemental Figure 4: Defective Arp2/3-mediated functions in a second ARPC1B-deficient patient. (A-D) Immunofluorescence and flow cytometry analysis of T cells from a second ARPC1B-deficient patient and a separate HD. (A) Co-staining with CD8 (green), ARPC1B (red) and phalloidin (white) showed absence of ARPC1B and altered morphology in hCTL from ARPC1B-deficient patient compared to HD. (B) Co-staining with CD8 (green), GLUT1 (red) and EEA1 (white) showed both membrane and intracellular localisation of GLUT1 in HD and a loss of membrane localisation of GLUT1 in ARPC1B-deficient patient hCTL. (C) Measurement of the mean intensity of GLUT1 expressed in AU (see methods) in HD and ARPC1B-deficient patient hCTL based on images as sampled in (B); p value < 0.05 (unpaired t-test). Images are 3D reconstruction of z-stacks. Scale bars: 4μm. (D) Density plot showing percentage of

CD3+CD8+ and CD3+CD4+ T cells in HD and ARPC1B-deficient patient PBMC, before (day 0) and after (day 7) stimulation with PHA and irradiated buffy coat (see methods).

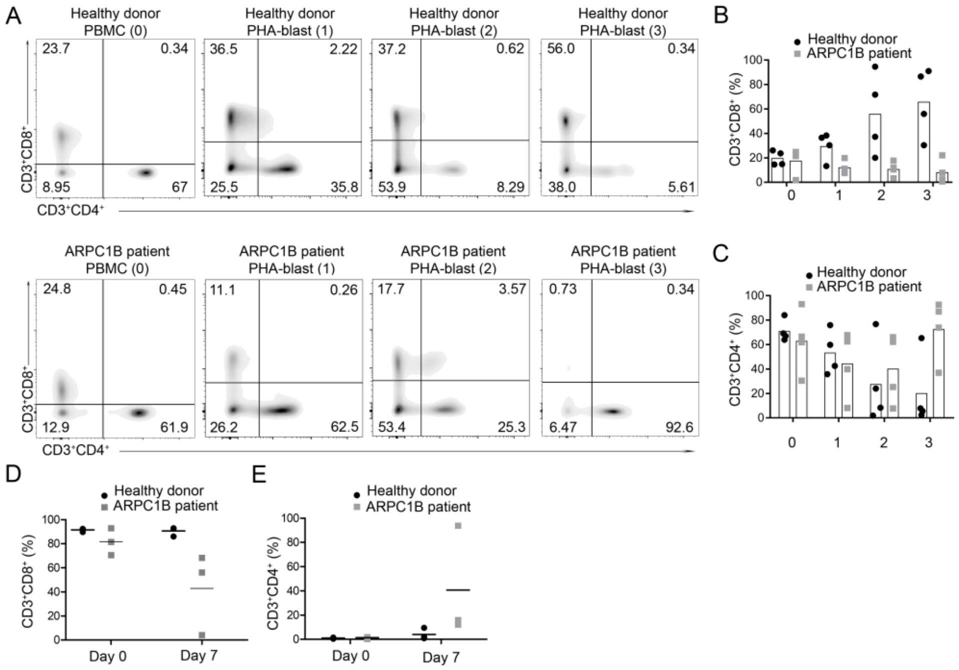

Supplemental Figure 5: Absence of ARPC1B affects CD8 T cell maintenance. (A) Representative density plots from one series of experiments showing the percentage of CD3+CD8+ and CD3+CD4+ T cells in PBMC from HD and ARPC1B-deficient patient before (left panels) and after 1, 2 or 3 stimulations with PHA and irradiated buffy coat (PHA-blast 1-3; see methods). (B and C) Summary of 4 separate time courses for stimulation showing percentage CD3+CD8+ (B) or CD3+CD4+ (C) from PBMC or PHA blasts (0) from HD (black circles) and ARPC1B-deficient cells (grey squares) after 1, 2 or 3 stimulations. n= 4 independent experiments. (D-E) Graph summarising 3 independent experiments showing purified CD3+CD8+ from HD and ARPC1B-deficient patient before (day 0) and after (day 7) stimulation with PHA and irradiated buffy coat (see methods). n= 3 independent experiments.

Supplement Video 1, related to Figure 3C: CK689 (control) treated OT-I CTL expressing EGFP-Lifeact (green, actin) and PACT-mRFP (red, centrosome) interaction with EL4 expressing Farnesyl-5-TagBFP2 (blue, target cell). Interval of each time point is 10sec and the duration of movie is 10min, x50 play speed.

Supplement Video 2, related to Figure 3C: CK666 treated OT-I CTL expressing EGFP-Lifeact (green, actin) and PACT-mRFP (red, centrosome) interaction with EL4 expressing Farnesyl-5-TagBFP2 (blue, target cell). Interval of each time point is 10sec and the duration of movie is 10min, x50 play speed.

Supplement Video 3, related to Figure 3D: Cell motility of the CK689 (control) treated OT-I CTL expressing EGFP-Lifeact. Interval of each time point is 3sec and the duration of movie is 5min, x60 play speed.

Supplement Video 4, related to Figure 3D: Cell motility of the CK666 treated OT-I CTL expressing EGFP-Lifeact. Interval of each time point is 3sec and the duration of movie is 5min, x60 play speed.

Supplemental Video 5, related to Figure 5C: HD hCTL expressing mApple-Lifeact-(red, actin) interaction with P815 (blue, target). Interval of each time point is 12sec and the duration of movie is 26min, x120 play speed.

Supplemental Video 6, related to Figure 5C: ARPC1B-deficient hCTL expressing expressing mApple-Lifeact (red, actin) interaction with P815 (blue, target). Interval of each time point is 12sec and the duration of movie is 26min, x120 play speed.
